# Effect of Verbal Instruction on Motor Learning Ability of Anaerobic and Explosive Exercises in Physical Education University Students

**DOI:** 10.3389/fpsyg.2019.02097

**Published:** 2019-09-25

**Authors:** Souhail Hermassi, Maha Sellami, El Ghali Bouhafs, René Schwesig, Andrea De Giorgio

**Affiliations:** ^1^Sport Science Program, College of Arts and Sciences, Qatar University, Doha, Qatar; ^2^Department of Sports Science, Prevention Rehabilitation, Martin-Luther-University Halle-Wittenberg, Halle, Germany; ^3^Department of Orthopaedic and Trauma Surgery, Martin-Luther-University Halle-Wittenberg, Halle, Germany; ^4^Department of Psychology, eCampus University, Novedrate, Italy

**Keywords:** physical education, feedback, motor learning, agility training, explosive performance

## Abstract

This study investigated the effect of motor learning with informational feedback into response to anaerobic exercises with and without motor learning tasks in handball physical education university students. Participants were randomly divided into two groups: experimental group (EG, *n* = 10) and control group (CG, *n* = 10). Measurements of T-half test, 15-m and 30-m sprints, and ZIG-ZAG test were assessed in both groups before (T1), between (T2) a 4-week intervention program, and after (T3) an 8-week intervention program, which included agility and speed teaching with (EG) or without (CG) informational feedback (i.e., verbal instruction). The test-retest reliability for all tests was excellent, and the ICC ranged from 0.76 (ZIG-ZAG test) to 0.99 (Agility *T* test). The interday measurement error was clearly below 1% in all tests (CV range: 0.2–0.8). Time effects for the Agility *T* test (*p* = 0.012, $\eta_{p}^{2}$ = 0.245) and the 15-m sprint (*p* = 0.035, $\eta_{p}^{2}$ = 0.190) were found. For the Agility *T* test, a total interaction effect (*p* = 0.001, $\eta_{p}^{2}$ = 0.380) and a partial interaction effect were calculated between T2 and T3 (*p* < 0.001, $\eta_{p}^{2}$ = 0.603). A large effect size (*d* = 0.87) was observed in the EG from T2 to T3. The second relevant (*d* ≥ 0.5) effect size was calculated for the parameter sprint 30 m. The CG showed a significant sprint performance reduction from T2 to T3 (*d* = −0.60; parameter: sprint 30 m). All other effect sizes were less than 0.44. The ZIG-ZAG test revealed the largest main and partial effect sizes for all parameters. The EG showed the largest improvement (*d* = 2.00) between T2 and T3. The results demonstrate that motor learning with informational feedback improves performances of Agility *T* test, sprint, and ZIG-ZAG performance. It appears that a well-formulated verbal instruction may induce performance enhancement in young trainees in educational environment.

## Introduction

In team sports, scientists have developed a lot of practical and learning techniques to provide educational and academic resources in order to obtain better knowledge among learners (Lauber and Keller, 2014; De Giorgio et al., 2018, 2019; Hraste et al., 2018). Several techniques and tools can be put at the service of sport practice and physical education, especially in motor learning (ML; Schmidt and Lee, 2011; Drost and Todorovich, 2017).

ML represents a recent concept (decade) involving several experiences that should be related to everyday life and alter the motor performance (Guthrie et al., 1952; Ugrinowitsch and Benda, 2011). Alteration of motor performance goes through internal process including cognitive process (brain and sensors stimulation) and allowing a person to change his/her behavior each time he/she is confronted with a task to which he/she does not yet have a suitable answer. This gradual change must be sustainable and create opportunities for skill development. It is necessary to take into account that ML could be linked to many factors belonging to the training process, such as verbal instruction and demonstration (Fagundes et al., 2013) or feedback (Chiviacowsky et al., 2009).

However, concerning the approaches for the most effective feedback, controversial findings are discussed (Drost and Todorovich, 2017). The demonstration method occurs the transfer of spatial and temporal movement information that allow the subject to develop a cognitive representation about the action. Obviously, the instruction methods seem to support improving responses to received tasks. On the other side, feedback strategies are very complex and can be classified based on the point in time at which feedback is provided: either during motor action task execution [i.e., concurrent (online, real time) feedback] or after it (i.e., terminal feedback; Lauber and Keller, 2014; Drost and Todorovich, 2017).

Moreover, feedback can be divided into general or informative/critique feedback, and both can be positive or negative (Fishwick et al., 1972; Koka and Hein, 2006). Previous studies also describe the so-called “feedback sandwich technique” (positive comment; critique; positive comment). For example, a positive comment in a basket such as “Good match today!,” a critique such as “Pay attention to your hand during throwing,” and a positive comment such as “Your match today was very good in person-to-person defense” (Lauber and Keller, 2014) elicits very different reactions in athletes.

Despite this complexity, feedback techniques are considered useful to improve ML (Koka and Hein, 2006; Lauber and Keller, 2014). Indeed, many studies highlighted the feedback effects on subjects’ behavior (Chiviacowsky et al., 2012), but so far as we know, no study has investigated the influence of verbal instructions regarding motor learning responses. Rarely, coaches and physical education specialist teachers take into account the feedback forms and effective moments of intervention. In fact, it seems that physical education teachers use an inappropriate feedback into the learning process. Consequently, the knowledge assimilation process will be negatively influenced (Nideffer, 1976).

Therefore, the main goal of the study was to investigate the effects of ML during an athletic activity in students. The principal question will be focused on evaluating the effect of the introduction of feedback during or at the end of the teaching of physical activity of agility and sprint performance in moderately trained athletes.

## Materials and Methods

### Participants

All participants were handball physical education university students. A written consent in this study was obtained from all participants after being thoroughly informed about the purpose, benefits, and potential risks of this experimental study. Consent forms were specifically approved by the “Research Unit Sport Performance, Health and Society: University of La Manouba” (the institutional review committee). This institutional review committee evaluated and approved the whole study design, which was conducted according to the ethical standards of the 1964 Helsinki Declaration and its subsequent amendments.

Before participation, a questionnaire was used to capture the following variables: age, height, body mass, medical history, training characteristics, performance level, and injury history. Furthermore, the team physician conducted an initial physical examination focused on orthopedic and other conditions that might preclude the spring and agility training.

Participants who met the inclusion criteria were randomly assigned to a control group (CG) or an experimental group (EG). Students participated in a physical education intervention provided by the Ministry of Higher Education and Scientific Research. The program included exercises from track and field (e.g., general and basic running technique, long jump exercises, 100-m running), gymnastics, and various team sports such as handball (e.g., pass, dribbling, control, throwing the ball), volleyball (e.g., passing, receiving, service), and soccer (e.g., pass, dribbling, control, shoot the ball). Each physical education session (total duration: 60 min) was performed in a similar way (standard warm-up, main session, and cool-down).

At the initial check of anthropometric and physical measurements, CG and EG were well matched in terms of physical characteristics (EG: age: 21.8 ± 0.5 years, body mass: 82.5 ± 5.8 kg, height: 1.80 ± 0.05 m, body fat: 13.4 ± 0.3%; CG: age: 22.1 ± 0.2 years, body mass: 83.2 ± 11.1 kg, height: 1.83 ± 0.03 m, body fat: 13.8 ± 0.1%).

### Motor Learning Intervention

This study utilized a longitudinal (two sessions), quasi-experimental design because of the desire to investigate the climate intervention in a real-world setting. Intact classes at each school were assigned to groups. The motor learning intervention training for EG started 1 week after baseline testing and consisted of two sessions weekly, continuing for 8 consecutive weeks consisting of 16 sessions. The motor skill program focused on multiple diagonal agility frontal and ZIG-ZAG sprinting. During the sprint and agility training period, the demands of the training sessions were progressively increased by decreasing the rest intervals between all sets of training. The intensity of training was individualized by instructing participants to perform a determined maximum number of repetitions per set of training (Supplementary Table S1). The maximum number of repetitions per set that each participant could perform during the workout period was established by an individualized test before the start of sprint and agility training. However, during all training sessions, maximal effort was encouraged verbally. The CG was given only general positive feedback (no informational/critique) such as “Well done” and “Good job” (Docheff, 1990; Lauber and Keller, 2014), and without any correction/support to movements. The provision of no feedback was strictly controlled (e.g., no facial expressions). On the contrary, the EG was given informational (i.e., critique) feedback into order to improve the self-awareness of participants on their movements; for example, we provided phrases such as “Your hand placement is perfect” or specified something corresponding to the task presentation that needed to be considered in future attempts: “Next time, slide step toward the target” (Drost and Todorovich, 2017). All sprint and agility training sessions started with a standard warm-up, consisting of 5 min of general dynamic exercises (low-intensity running, high skipping, leg flexions, lateral running, front and behind arm rotation, and sprints). Before all training sessions, participants performed three sets, with 30 and 20 repetitions of physical exercises involving the lumbar muscular and abdominal groups.

### Testing Schedule

Three similar sets of tests were planned and integrated into the weekly training schedules. All measurements were performed on a regular indoor court, under similar conditions (temperature 20.5 ± 0.5°C; relative humidity 60 ± 5%) and at the same time of day (5:00 to 7:00 p.m.). To prevent effects of fatigue, intensive physical training was avoided for 24 h prior to the test. Participants also fasted for at least 3 h before the investigation. A standardized battery of warm-up exercises (5 min of low-intensity running, two sets of 3 m × 30 m progressive accelerations, and two sets of maximal 30-m sprint, interspersed with 2- to 3-min periods of passive recovery) preceded all maximal efforts. The first set of tests, completed 2 weeks before the intervention, familiarized participants with the testing procedures. Furthermore, we were able to evaluate the test-retest reliability of measurements. The second test was given between the intervention, and the third test session was conducted immediately following the intervention.

### 15-m and 30-m Sprints

Sprint testing began with standardized dynamic warm-up (~10 min) followed by sub-maximal 30-m shuttle runs (intensity: 60–70% of maximum heart rate) and four acceleration sprints during the runs. From a standing position, subjects ran 40 m, with the front foot 0.2 m behind the starting photocell beam. However, times at 15 and 30 m were recorded by paired photocells (Microgate, Bolzano, Italy) that were located 1 m above the ground at the starting and finishing lines. Three trials were separated by 6–8 min of recovery in order to avoid fatigue effects of evaluation. The fastest time was used for the analyses.

### Ability to Change Direction (*T*-Half Test)

A 15-min warm-up included running, jogging, lateral displacements, dynamic stretching, and vertical jumping. The Standard *T*-half tests (Sassi et al., 2009) were performed in a random order. The total distance covered was reduced from 36.56 to 20 m. Data were recorded using an electronic timing system (Globus, Microgate SARL, Bolzano, Italy). Electronic timing sensors were set 0.75 m above the floor, 3 m apart, and facing each other at the starting line (Sassi et al., 2009). The testing started with both feet placed behind the starting line A. Subjects sprinted forward to cone B and touched its base with their right hands. Facing forward and without crossing feet, they then shuffled to the left to cone C and touched its base with their left hands. Afterward, they shuffled to the right to cone D and touched its base with their right hands, subsequently shuffling back to the left to cone B and touching its base. Finally, they ran backward as quickly as possible, returning to line A. Anyone who crossed one foot in front of the other and failed to touch the base of a cone and/or failed to face forward throughout had to repeat the test (rest time between trials: 3 min). Criteria for an acceptable test were as in the *T*-half test, with the better of two definitive trials recorded (Sassi et al., 2009).

### ZIG-ZAG Test

The ZIG-ZAG test course consisted of four 5-m running sections set at 1008 angles. The test was chosen because it requires the short acceleration, deceleration, and balance control facets of agility. The familiarity of the participants with the ZIG-ZAG test and its relative simplicity minimized learning effects. The timing began on a sound signal and stopped when the subject passed through a timing gate.

### Training Intervention Program

The EG performed two workouts per week, in addition to their usual physical education requirements, for 8 weeks. The EG regimen consisted of 2 sessions/weeks of agility and sprint training. The training program was based on recommendations of Young et al. (2001) using similar drills. From a psychological and physiological point of view, 4–8 weeks of training is an optimal time interval for adaptation to be stressed without excessive strain or fatigue (Young et al., 2001). It is believed that neuromuscular adaptations contributing to explosive power of lower limb occur early in the power cycle of the periodization phase of physical training (Young et al., 2001). Agility and sprint training was only performed twice per week to allow for sufficient recovery between workouts as recommended by researchers (Young et al., 2001). The subjects were instructed to use a “complete” recovery between sprints, agility training, and ZIG-ZAG sprint (typically 2–5 min) and to avoid any worsening of times as the session progressed. The length of each interval varied from 15 to 30 m to provide variety and allow an individual sprint and agility training. The average of intensity was slightly below the maximum speed for the first 2 weeks to allow progression and to reduce the risk of injury. The intensity of the submaximum efforts was monitored by providing feedback to each participant on how his/her physical interval time compared with the times achieved during the pretesting.

### Statistical Analyses

All statistical analyses were performed using SPSS version 25.0 for Windows (SPSS Inc., Chicago, IL, USA). Descriptive statistics (mean, standard deviation, 95% confidence interval) were ascertained for all parameters. Differences between groups (EG vs. CG) and sessions (examination 1 vs. examination 2 vs. examination 3) were tested using a two-factor (time, group) univariate general linear model (Bortz, 1999).

The intrarater reliability was evaluated using the first two sessions. Intraclass correlation coefficients (ICCs) and coefficients of variation (CVs) were calculated, with interpretation as proposed by Portney and Watkins (2009). An ICC >0.75 was rated excellent, coefficients between 0.40 and 0.75 were rated fair to good, and values <0.40 were rated poor (Shrout and Fleiss, 1979). The CV, an indicator of measurement variability and random error, was derived from log-transformed data (Hopker et al., 2010). The CV is a reliability measure with ≤10% commonly used as a criterion to characterize good reliability (Brughelli and Van Leemputte, 2013).

The effect size (*d*; mean difference of scores divided by the pooled standard deviation) was calculated for each parameter (Hartmann et al., 1992; Rhea, 2004). A positive effect size implies an improvement of performance and a negative value indicates a deterioration in performance. Partial eta squared ($\eta_{p}^{2}$) was calculated for the ANOVA main effects (Richardson, 2011) and used to estimate the practical relevance of the performance differences. The significance criteria for mean differences (group, time, and group-time effects) were *p* < 0.05 and $\eta_{p}^{2}$ > 0.20 and *d* ≥ 0.5 and Δ*d* ≥ 1.0.

## Results

### Reliability

The test-retest reliability for all tests was excellent (Supplementary Table S2). The ICC ranged from 0.76 (ZIG-ZAG test) to 0.99 (Agility *T* test). The interday measurement error was clearly below 1% in all tests (CV range: 0.2–0.8).

### Performance Analysis

**Figure 1:**
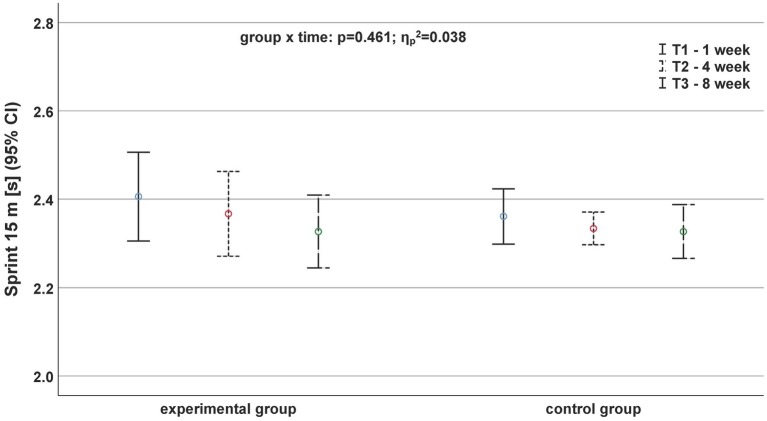
Development of sprinting performance based on the parameter sprint 15 m before (continuous black line), after 4 weeks (fine dashed black line), and after (roughly dashed black line) the exercise program depending on the group.

**Figure 2:**
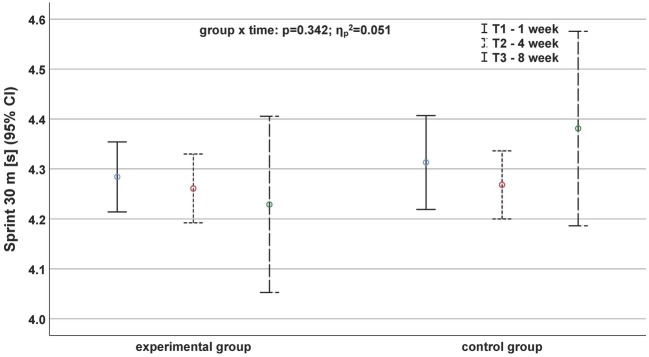
Development of sprinting performance based on the parameter sprint 30 m before (continuous black line), after 4 weeks (fine dashed black line), and after (roughly dashed black line) the exercise program depending on the group.

Regarding the sprinting performance (Figures 1, 2), we did not observe any significant interaction effect. Only a time effect for the 15-m sprint (*p* = 0.035, $\eta_{p}^{2}$ = 0.190) was detected.

Only one relevant (*d* ≥ 0.5) effect size was found during the 30-m sprint. The CG showed significantly slower 30-m sprint times between T2 and T3 (*d* = −0.60). All other effect sizes for sprinting parameters were less than 0.44.

**Figure 3:**
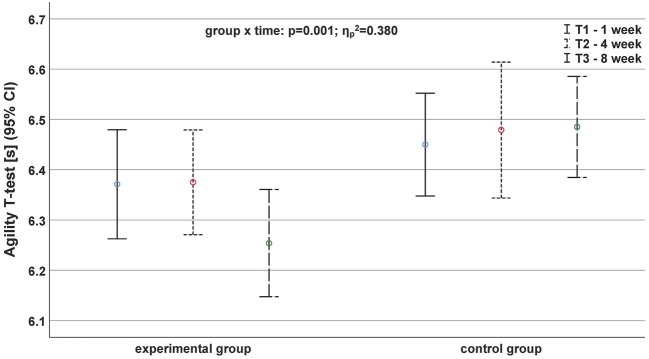
Development of agility performance based on the parameter agility *T*-test before (continuous black line), after 4 weeks (fine dashed black line), and after (roughly dashed black line) the exercise program depending on the group.

**Figure 4:**
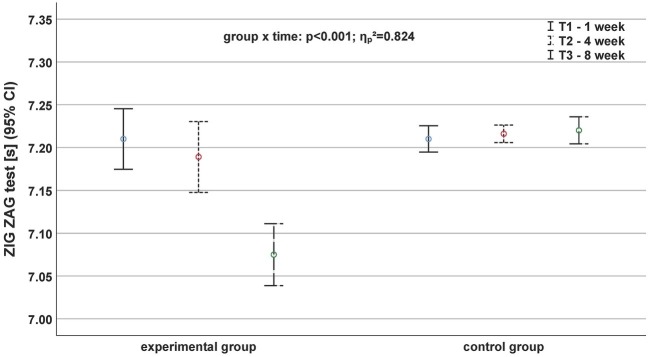
Development of agility performance based on the parameter ZIG-ZAG test before (continuous black line), after 4 weeks (fine dashed black line), and after (roughly dashed black line) the exercise program depending on the group.

In contrast to the sprinting performance, we found significant main time and interaction effects for the agility parameters (Supplementary Table S3). The Agility *T* test (*p* = 0.001, $\eta_{p}^{2}$ = 0.380; Figure 3) and the ZIG-ZAG test (*p* < 0.001, $\eta_{p}^{2}$ = 0.824; Figure 4) displayed significant inter-group differences.

A partial interaction effect was detected for the Agility *T* test between T2 and T3 (*p* < 0.001, $\eta_{p}^{2}$ = 0.603; Figure 3). In this context, the EG showed a large effect size (*d* = 0.87) between T2 and T3. In contrast, the first period (T1 to T2) showed a small reduction of agility performance (*d* = −0.07; 6.37 ± 0.15 vs. 6.38 ± 0.15 s). For Agility *T* test, we only observed negative effect sizes for both groups in the first period (*d*_EG_ = −0.07; *d*_CG_ = −0.18). In contrast, the effect sizes in the second period (T2 to T3) increased from −0.06 (CG) to 0.87 (EG).

The ZIG-ZAG test revealed the largest main and partial effect sizes for all parameters (Figure 4). The EG showed the largest improvement (*d* = 2.00) between T2 and T3 (Supplementary Table S3). Comparable with the Agility *T* test, the CG showed a reduction of performance between T1 and T2 (*d* = −0.67) and did not show any change of performance (*d* = 0) during the second period (T2 to T3).

## Discussion

The results of this study showed that students exposed to both motor learning and informational (i.e., critique) feedback increased their performance on anaerobic and explosive exercises compared to the CG, which showed no significant gains in the proposed exercises. The current study seems to improve the scientific background in terms of informational feedback effects on anaerobic exercises using short (15 m) and mid sprint (30 m), ZIG-ZAG, and agility test (*T*-half test).

How the feedback is delivered and the way in which students responded to this feedback would allow one to give an important novel idea about the information and motor learning. As we know, feedback effect is crucial for learning and can reduce both cognitive load and uncertainty about performance. Moreover, feedback highlights next steps for reaching learning goals (Hattie and Timperley, 2007; Shute, 2008). Therefore, clear and specific feedback can give crucial information on the current task to the learner in such a way that it is possible to guide the person toward following steps (Hattie and Timperley, 2007; Shute, 2008). Conversely, it has been demonstrated that discouraging feedback can influence and reduce both performance and learning (Hattie and Timperley, 2007; Shute, 2008). Climate is also very important and literature highlights that positive comments and praise – particularly from peers – are considered especially necessary (Shute, 2008).

Moreover, Chiviacowsky et al. (2012) demonstrated that the amount of correct practice trials that a physical student accumulates is a determinant for learning. In line with Chiviacowsky et al. (2012), although time on task and practice trials was not calculated in this study, we speculate that physical students in the EG could spend more time on tasks and could persist longer on tasks by informational feedback when they experienced difficulty, thus helping to improve their motor skill performance. Future research designs in mastery climate settings would benefit by accounting for the time children spend practicing different motor skills.

This investigation also showed that subjects in the EG increased their performance on anaerobic and explosive exercises during the intervention of training period. In contrast, the CG showed no significant gains compared with the EG. Sprinting, the ability to make rapid changes in direction and acceleration, is an important quality for students of physical education (Mekota et al., 2012). We can assume from our results that the quality of feedback was able to improve sprint times (i.e., faster following the program training; Supplementary Table S1), and this is also in accordance with previous investigation (Mekota et al., 2012) where the 5- and 20-m sprint times of healthy young males were improved after 12 weeks of training.

It has been demonstrated that giving feedback to motor task could enhance performance during multiple complex movements (Fortier et al., 2005). Therefore, for practical application, the sprint coaches should provide feedback into their training sessions to refine athletes’ movement patterns. Note that current results have proven that the method used improved performance in sprint times (i.e., faster following the program training; Supplementary Table S1).

Results from literature confirmed the effectiveness of the feedback during multiple task movements (Fortier et al., 2005), while other studies show a low effectiveness if this method is used to enhance performance skill acquisition (Wulf et al., 1999). Also, it has been suggested that observational learning is sometimes sufficient to allow the development of an error detection mechanism necessary for improving sprint performance (Blandin and Proteau, 2000). In our experiment, the subjects were taught to use feedback (i.e., specific instructions) to gain control over their response patterns. In fact, the standard deviation of 30-m sprint times of both EG and CG at 8 weeks seems to be larger than at 1 and 4 weeks. This can be probably explained by the effect of the individual assimilation of students related to feedback during the training sessions. Moreover, to the best of our knowledge, the present study seems to be the first to have examined the effects of informational/critique feedback upon the agility of physical education students.

Likewise, a global interaction effect (*p* = 0.001, $\eta_{p}^{2}$ = 0.380) was only found for the Agility *T*-half test. In addition, a partial interaction effect was also detected for the Agility *T* test from T2 to T3 (*p* < 0.001, $\eta_{p}^{2}$ = 0.603). In this context, the EG showed a large effect size (*d* = 0.87) from T2 to T3.

The present study sheds new light on the role of feedback for motor learning. Indeed, literature demonstrated that feedback for motor learning can also be effective when performers’ attention is moved away from their body movements (external feedback; Wulf et al., 2002; De Giorgio et al., 2019). Verbal instructions vs. external feedback are two different approaches, each one having its own particularity but the current predominant observation here is that “feedback manipulations” seem to be more effective when it comes to enhancing the learners’ awareness of their body movements (Schmidt, 1991; Schmidt and Lee, 1999; Wulf et al., 2002). In fact, conscious control of movements is assumed to be essential for learning especially early in the learning process (Fitts, 1964; Adams, 1971).

The performances of the ZIG-ZAG test revealed the largest main and partial effect sizes for all parameters. The EG showed the largest improvement (*d* = 2.00) between T2 and T3. According to that view, the effectiveness of feedback is enhanced to the extent that it encourages participant learners, or at least gives learners a chance, to attend to their own physical movements (Salmoni et al., 1984; Schmidt, 1991). For example, beyond motor sports skills, some scientific studies have examined the role of feedback into the physical performance and learning of surgical skills, such as suturing or knot tying (Stefanidis et al., 2007; O’Connor et al., 2008). Instructor feedback into medical training is quite obvious, but can make us understand well how investigations should take into account the frequency and type of feedback. Therefore, the specificity and/or the interactive effects on the motor learning skills can be well assessed. In addition, it has been shown that motivation plays an important role during feedback and, therefore, motor learning. In their study, Chiviacowsky and Wulf (2007) suggested that giving learners explanation after “good” trials, compared to after “poor” trials, results in more effective learning.

The current study confirms that students exposed to both motor learning and informational (i.e., critique) feedback increased their performance on anaerobic and explosive exercises compared to the CG, which showed no significant gains in the proposed exercises. Actual findings would allow a novelty in teaching motor learning process during physical activity.

### Limitations

Current findings should be interpreted and used with caution since results are based on a small sample size and slight differences between groups were recorded in the initial comparison.

In addition, it is important to extend investigation to players with different categories of age and gender to confirm the effectiveness of this method. Further, there is a need to assess gains in terms of anaerobic exercises with and without motor learning tasks based on the number of the feedback into all training sessions.

## Conclusion

The current study confirmed the important role of feedback during motor learning. It suggested that feedback manipulation is the best way to enhance body awareness during movements. However, further studies are necessary to evaluate the frequency and type of feedback well in order to define the specific or interactive effects on motor learning skills.

## Data Availability Statement

The datasets for this manuscript are not publicly available because the datasets for this manuscript are not publicly available because of legal reasons. Requests to access the datasets should be directed to the corresponding author.

## Ethics Statement

Consent forms were specifically approved by the “Research Unit Sport Performance, Health and Society: University of La Manouba” (The institutional review committee).

## Author Contributions

All authors listed have made a substantial, direct and intellectual contribution to the work, and approved it for publication.

### Conflict of Interest

The authors declare that the research was conducted in the absence of any commercial or financial relationships that could be construed as a potential conflict of interest.
