# Supplementary material for: Effect of Verbal Instruction on Motor Learning Ability of Anaerobic and Explosive Exercises in Physical Education University Students
Source: Front Psychol. 2019 Sep 25;10:2097. doi: 10.3389/fpsyg.2019.02097 (PMC6798030; doi:10.3389/fpsyg.2019.02097)
Supplement: Supplementary file 1 [file Table_1.docx]

**Table 1.** Training program for agility and sprint-training program over 8 weeks.

|  | Frontal | | Multiple diagonal | Zig Zag sprint | | Frontal |
| --- | --- | --- | --- | --- | --- | --- |
|  | Sprint 15 m | | Agility | with 110° curves | | Sprint 30 m |
| Set x repetition | S x R (m) | | S x R (%) | S x R (m) | | S x R (m) |
| Session interval |  | |  |  | |  |
| Session 1 | 2 x 8: 15 | | 3 x 6: 5*5*5 | 3: 4 × 5 | | 2 x 8: 30 |
| Session 2 | 2 x 8: 15 | | 3 x 6: 5*5*5 | 3: 4 × 5 | | 2 x 8: 30 |
| Session 3 | 2 x 8: 15 | | 3 x 6: 5*5*5 | 3: 4 × 5 | | 2 x 8: 30 |
| Session 4 | 2 x 8: 15 | | 3 x 6: 5*5*5 | 3: 4 × 5 | | 2 x 8: 30 |
| Session 5 | 2 x 8: 15 | | 3 x 6: 5*5*5 | 3: 4 × 5 | | 2 x 8: 30 |
| Session 6 | 2 x 8: 15 | | 3 x 6: 5*5*5 | 3: 4 × 5 | | 2 x 10: 30 |
| Session 7 | 2 x 10: 15 | | 3 x 6: 5*5*5 | 3: 6 × 5 | | 2 x 10: 30 |
| Session 8 | 2 x 10: 15 | | 3 x 6: 5*5*5 | 3: 6 × 5 | | 2 x 10: 30 |
| Session 9 | 2 x 10: 15 | | 3 x 6: 5*5*5 | 3: 6 × 5 | | 2 x 10: 30 |
| Session 10 | 2 x 10: 15 | | 3 x 6: 5*5*5 | 3: 8 × 5 | | 2 x 10: 30 |
| Session 11 | 2 x 10: 15 | | 3 x 6: 5*5*5 | 3: 8 × 5 | | 2 x 10: 30 |
| Session 12 | 2 x 10: 15 | | 3 x 6: 5*5*5 | 3: 8 × 5 | | 2 x 10: 30 |
| Session 13 | 2 x 12: 15 | | 3 x 6: 5*5*5 | 3: 8 × 5 | | 2 x 12: 30 |
| Session 14 | 2 x 12: 15 | | 3 x 6: 5*5*5 | 3: 8 × 5 | | 2 x 12: 30 |
| Session 15 | 2 x 12: 15 | | 3 x 6: 5*5*5 | 3: 8 × 5 | | 2 x 12: 30 |
| Session 16 | 2 x 12: 15 | | 3 x 6: 5*5*5 | 3: 10 × 5 | | 2 x 12: 30 |
|  |  | 2 sets with 5 min rest | | |  |  |

**Table 2.** Comparison of test measurements obtained from the two testing sessions for handball players (n=20). Descriptive statistics (mean ± SD) and intrarater reliability analysis calculated for each parameter are presented. ICC ≥ 0.75 and CV ≤ 5% are highlighted in bold.

| **test** | **Session one** | **Session two** | **ICC** | **CV (%)** |
| --- | --- | --- | --- | --- |
|  | mean ± SD | mean ± SD | (95% CI) | (95% CI) |
| **Sprint 15 m [s]** | 2.39 ± 0.12 | 2.41 ± 0.12 | **0.98 (0.90 – 0.99)** | **0.8 (0.6 – 1.2)** |
| **Sprint 30 m [s]** | 4.30 ± 0.11 | 4.32 ± 0.11 | **0.98 (0.74 – 0.99)** | **0.4 (0.3 – 0.6)** |
| **Agility T-test [s]** | 6.41 ± 0.15 | 6.43 ± 0.15 | **0.99 (0.74** – **1.00)** | **0.2 (0.1 – 0.2)** |
| **ZIG-ZAG test [s]** | 7.21 ± 0.04 | 7.24 ± 0.03 | **0.76 (0.00 – 0.94)** | **0.2 (0.1 – 0.2)** |

**Table 3.** Comparison of sprint parameters between experimental group (EG) and control group (CG). Values are given as mean ± SD. d=effect size. Significant effects (p<0.05) are highlighted in bold.

|  | **EG (n=10)** | | | **CG (n=10)** | | | **Variance analysis/ effects p (η_p_^2^)** | | | | |
| --- | --- | --- | --- | --- | --- | --- | --- | --- | --- | --- | --- |
|  | T1 – 1 week | T2 – 4 week | T3 – 8 week | T1 – 1 week | T2 – 4 week | T3 – 8 week | group | time | | group x time | |
| **Agility and Sprinting performance** [s] | | | | | | | | | | | |
| **Sprint 15 m** | 2.41 ± 0.14 | 2.37 ± 0.13 | 2.33 ± 0.12 | 2.36 ± 0.09 | 2.33 ± 0.05 | 2.33 ± 0.09 | 0.544  (0.021) | **0.035 (0.190)** | | 0.461  (0.038) | |
| Partial  effect sizes and  group x time effects | d_1/2_=0.30 d_2/3_=0.32 | | | d_1/2_=0.43 d_2/3_=0 | | | T1/T2: p=0.694; η_p_^2^=0.009 T2/T3: p=0.373; η_p_^2^=0.044 | | | | |
| **Sprint 30 m** | 4.28 ± 0.10 | 4.26 ± 0.10 | 4.23 ± 0.25 | 4.31 ± 0.13 | 4.27 ± 0.10 | 4.38 ± 0.27 | 0.156 (0.109) | 0.599 (0.016) | | 0.342  (0.051) | |
| Partial  effect sizes and  group x time effects | d_1/2_=0.20 d_2/3_=0.17 | | | d_1/2_=0.35 d_2/3_=**-0.60** | | | T1/T2: p=0.350; η_p_^2^=0.049 T2/T3: p=0.294; η_p_^2^=0.061 | | | | |
| **Agility T-test** | 6.37 ± 0.15 | 6.38 ± 0.15 | 6.25 ± 0.15 | 6.45 ± 0.14 | 6.48 ± 0.19 | 6.49 ± 0.14 | 0.051  (0.196) | **0.012 (0.245)** | | **0.001  (0.380)** | |
| Partial  effect sizes and  group x time effects | d_1/2_=-0.07 d_2/3_=**0.87** | | | d_1/2_=-0.18 d_2/3_=-0.06 | | | T1/T2: p=0.534; η_p_^2^=0.022 T2/T3: **p<0.001; η_p_^2^=0.603** | | | | |
| **ZIG-ZAG** | 7.21 ± 0.05 | 7.19 ± 0.06 | 7.08 ± 0.05 | 7.21 ± 0.02 | 7.22 ± 0.01 | 7.22 ± 0.02 | **0.003  (0.403)** | | **p<0.001**  **(0.786)** | | **p<0.001**  **(0.824)** |
| Partial  effect sizes and  group x time effects | d_1/2_=0.36 d_2/3_=**2.00** | | | d_1/2_=-0.67 d_2/3_=**0** | | | T1/T2: **p=0.024**; **η_p_^2^=0.254** T2/T3: **p<0.001; η_p_^2^=0.850** | | | | |
